# Valproic acid determination by liquid chromatography coupled to mass spectrometry (LC–MS/MS) in whole blood for forensic purposes

**DOI:** 10.1002/dta.3362

**Published:** 2022-09-02

**Authors:** Jennifer P. Pascali, Arianna Giorgetti, Rossella Barone, Guido Pelletti, Paolo Fais

**Affiliations:** ^1^ Department of Cardiac, Thoracic, Vascular Sciences and Public Health University of Padova Padova Italy; ^2^ Department of Medical and Surgical Sciences, Unit of Legal Medicine University of Bologna Bologna Italy

**Keywords:** adducts, forensic toxicology, LC–MS, therapeutic drug monitoring, valproic acid

## Abstract

Valproic acid (VPA) is a well‐known drug prescribed as anti‐epileptic. It has a narrow therapeutic range and shows great individual differences in pharmacodynamics and pharmacokinetics. Consequently, the therapeutical drug monitoring (TDM) in patient's plasma is of crucial importance. Liquid chromatography coupled to mass spectrometry (LC–MS/MS) has gained importance in TDM applications for its features of sensitivity, selectivity and rapidity. However, in case of VPA, the LC–MS/MS selectivity could be hampered by the lack of a sufficient number of multiple reaction monitoring (MRM) transitions describing the molecule. In fact, the product ion scan of deprotonated molecules of VPA does not produce any ion and thus most LC–MS/MS methods are based on the detection of the unique MRM transition *m*/*z* 143➔143. In this way, the advantages of selectivity in LC–MS cannot be effectively exploited. In the present method, stable analyte adducts were exploited for the determination of VPA in blood. An Acquity HSS C18 column and mobile phases consisting of 5‐mM ammonium formate and acetonitrile both added 0.1% formic acid were used. Source worked in negative acquisition mode and parameters were optimized to increase the adduct (*m*/*z* 189) and dimer (*m*/*z* 287) stability, and their fragmentation were used to increase the selectivity of MRM detection. The method has been validated according to the toxicological forensic guidelines and successfully applied to 10 real blood samples. Finally, the present method showed suitable for the rapid LC–MS/MS detection of VPA in whole blood, demonstrating the possibility to increase specificity by exploiting stable in‐source adducts. This should be considered of utmost importance in the case of forensic applications.

## Graphical abstract

1. VPA adducts and dimers showed stable and reproducibly during method validation on blood.
2. The method proved suitable for VPA determination in cases of forensic interest

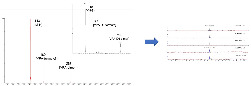

## 1 INTRODUCTION

Valproic acid (2‐propylpentanoic acid or VPA) is a short‐chain fatty acid derivative prescribed as anti‐epileptic, mood stabilizer, and for migraine prophylaxis.^1^ Moreover, VPA is used in the treatment of alcohol and substance withdrawal and dependence.^2^

It is commonly administered as sodium salt, sodium valproate, or a mixture of its acidic form and salt, showing high oral bioavailability and high binding to plasma proteins, with a plasma/whole blood ratio around 1.8.^3^ VPA has a narrow therapeutic range (50–100 μg/ml) and shows great individual differences in pharmacodynamics and pharmacokinetics. Consequently, the therapeutical drug monitoring (TDM) in patient's plasma is of crucial importance. From the analytical point of view, many methods for VPA determination in plasma exist in literature, and beside immunometric methods commonly used in clinical settings, high‐performance liquid chromatography (HPLC‐UV)^4^ and gas chromatography–mass spectrometry (GC–MS)^5^ have been gaining importance in recent years. Moreover, liquid chromatography coupled to mass spectrometry (LC–MS/MS)^6^, ^7^, ^8^ has gained importance in TDM method development for its features of sensitivity, selectivity, and rapidity. However, in case of VPA determination, the LC–MS/MS selectivity could be hampered by the lack of a sufficient number of multiple reaction monitoring (MRM) transitions describing the molecule. In fact, the product ion scan of deprotonated molecules of VPA does not produce any ion, and thus most LC–MS/MS methods are based on the detection of the unique MRM transition m/z 143➔143.^6^, ^9^, ^10^ In this way, the advantages of MRM detection cannot be effectively exploited. To overcome this limitation, adducts formation between VPA and mobile phase components as acetate and acetic acid have been described.^10^, ^11^ Adduct formation is based on complicated equilibria reactions at the source level, and these adducts, if noted, are usually not considered because of low reproducibility. However, some authors^12^ demonstrated the possibility to increase LC–MS/MS selectivity in case of analysis of tricky small molecules in biological matrices by considering ion adducts with mobile phase and their fragmentation pattern. This proved certainly useful for clinical applications, and also forensic cases^13^, ^14^ (i.e., fatal intoxication) would benefit by an increase of selectivity. In this view, we studied the possibility for VPA to form stable and reproducible adducts with formate, frequently adopted in LC–MS/MS mobile phases for multi‐target methods for determination of pharmaceutical and abused drugs. In these conditions, the VPA dimer adduct was observed and exploited to develop and validate a new rapid LC–MS/MS method. Finally, we applied the method to forensic cases by analyzing samples of whole blood. In fact, methods developed for TDM, which are based on plasma/serum material, may not be suitable for forensic purposes, because forensic samples are generally hemolyzed and plasma/serum is not available.

## 2 MATERIALS AND METHODS

### 2.1 Materials

Valproic acid sodium salt (VPA) and Valproic acid‐D6 (internal standard [IS]) were purchased by Merck (Darmstadt, Germany). Formic acid was acquired from Merck Millipore (Darmstadt, Germany). Ammonium formate for LC–MS was procured by Sigma–Aldrich (Milan, Italy). Acetonitrile and methanol (LC–MS grade) were supplied by Merck (Darmstadt, Germany). Water for mobile phase preparation was produced by Elga Veolia instrumentation (Lane End, High Wycombe, UK).

Stock solutions of VPA were prepared in methanol. Internal standard working solution was prepared at the final concentration of 20 μg/ml in methanol. All solutions were stored at −20°C and left at room temperature 2 h for equilibration prior use.

### 2.2 Sample preparation

Samples were prepared by spiking 20 μl of IS solution to 200 μl of blood in a microcentrifuge vial and adding 400 μl of cold acetonitrile. Samples were then vortexed for 10″ and centrifuged for 10′ at 12,000 rpm. Four hundred microliters of supernatant was transferred into a glass vial and taken to dryness at 40°C under a flux of nitrogen. Samples were reconstituted in 400 μl of water and injected into the LC–MS/MS system. Blank venous whole blood from healthy volunteers working in the laboratory was collected in ethylenediaminetetraacetic acid tubes (BD Vacutainer®) and used for the preparation of calibrators and quality control (QC) samples.

The method was applied to blood samples of 10 real forensic cases selected among deceased subjects under treatment with VPA, or samples collected in the frame of driving under the influence of drugs or judicial autopsy cases. Samples were from the laboratory repository, and time storage was between 1 week and 12 months. All samples were analyzed in duplicate, and the mean concentration was reported.

### 2.3 LC–MS conditions

The LC–MS system consisted of an UHPLC Water Acquity coupled to a Xevo TDQ triple quadrupole mass spectrometer (Waters, Milford, MA), equipped with an Acquity UPLC HSS C18 column (2.1× 150 mm, 1.8 μm) (Waters, Milford, MA) set at the temperature of 45°C. The optimized mobile phase consisted of water added with 5‐mM ammonium formate and 0.1% formic acid (mobile phase A) and 0.1% formic acid in acetonitrile (mobile phase B); flow rate was set at 0.3 ml/min. The optimized gradient was as follows: 20% B for 0.5 min, increased to 85% B from 0.5 to 7 min, increased to 95% from 7 to 7.5 min, held constant for 1.5 min, then decreased to initial condition of 20% in 0.1 min, and held until 11 min for equilibration. The volume of injection was 10 μl. Sample tray temperature was set at 10°C. The system was equipped with strong and weak wash solution reservoirs composed of a mixture of 50/40/10 (*v*/*v*) methanol, isopropanol, acetic acid, and 50/50 (*v*/*v*) water/acetonitrile. Washing times were set at 10 s each.

The triple quad instrument was operating in negative ion mode with the following optimized MS settings to maximize VPA‐adducts formation: capillary and cone voltage at 3 kV and 15 V, respectively, source temperature 150°C, desolvation temperature at 350°C, and nitrogen gas flow at 650 L/h. Optimized MRM transition are reported in Table 1.

**TABLE 1:** MRM transitions of valproic acid (VPA) and internal standard (VPA‐D6)

Analyte	Retention time (min)	Parent ion (*m*/*z*)	Fragment ion (*m*/*z*)	Cone (V)	Collision energy (V)
VPA	5.78	143	143	15	2
		189	143	15	10
		287	143	15	10
VPA‐D6	5.75	149	149	15	2
		195	149	15	10

*Note*: MRM, multiple reaction monitoring.

### 2.4 Method validation

The method was validated following the forensic toxicology guidelines by considering selectivity, sensitivity, linearity accuracy, precision, matrix effect, and stability.^15^ Selectivity was assessed by analyzing eight drug‐free blood samples, six without IS and two with IS. Matrix‐matched calibration curve was set in the range of 5–800 μg/ml (5–10–50–100–200–400–600–800 μg/ml). Three QCs at 25–75–500 μg/ml were also prepared in whole blood. Accuracy and precision were evaluated by analyzing QC samples in five replicates on four non‐consecutive days. Accuracy and precision were obtained as bias (%) and relative standard deviation (RSD %). Sensitivity, expressed as limit of detection (LOD) and limit of quantification (LOQ), was determined by a signal‐to‐noise ratio of 3 and 10, respectively, and experimentally verified by spiking the calculated amount. To obtain an average evaluation of the matrix effect over the entire quantification range, expressed as average bias %, the slopes of the calibration curves prepared in water and blood (n. 10 samples) were used.^16^, ^17^ Stability was assessed on QCs after 24 h storage at −20°C and calculating percent deviation on freshly prepared QCs.

## 3 RESULTS AND DISCUSSION

**FIGURE 1:**
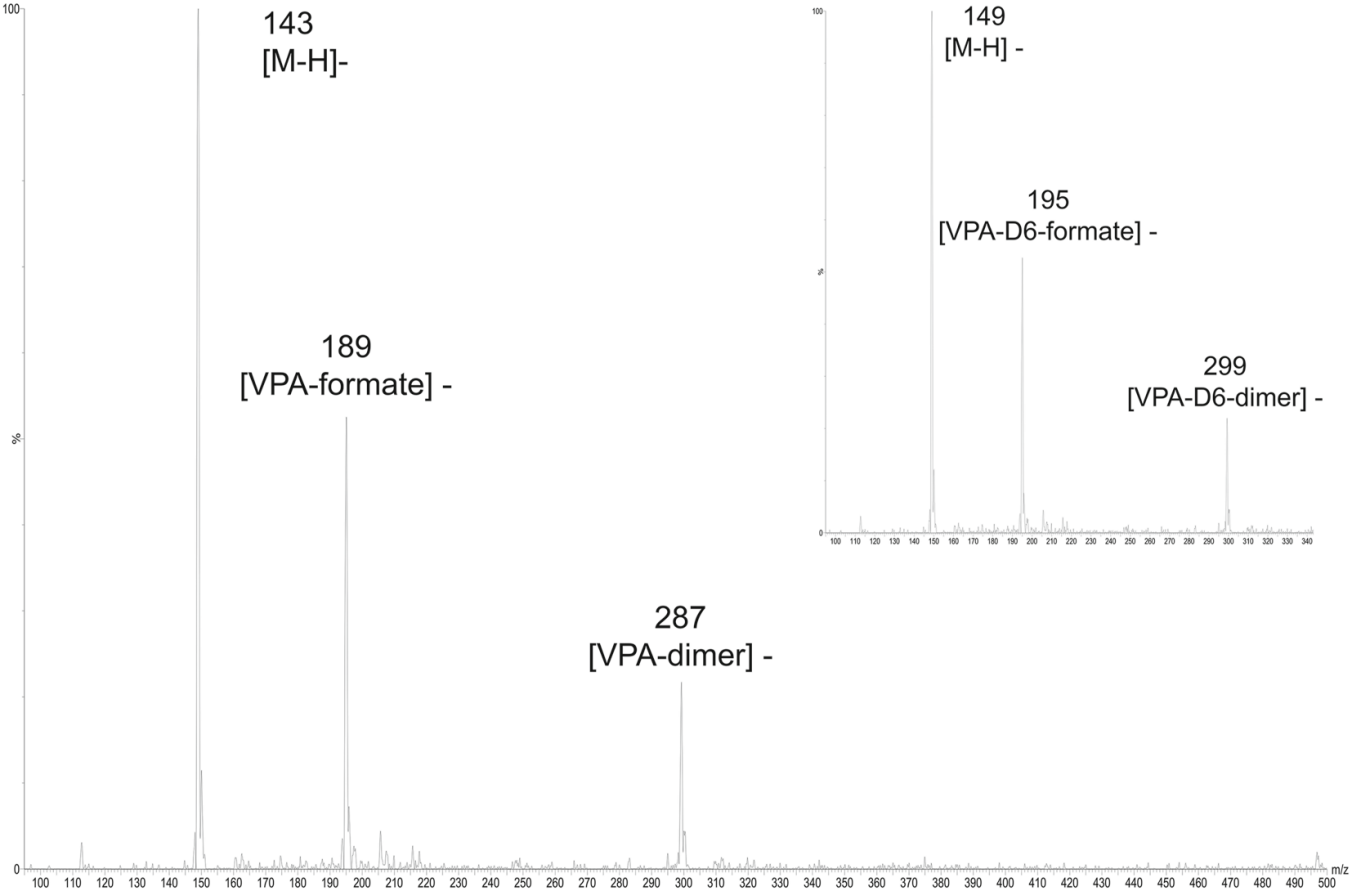
Background subtracted *m*/*z* spectra of valproic acid and valproic acid‐D6 standard solution in SCAN acquisition. Formate adducts and dimers are visible

**FIGURE 2:**
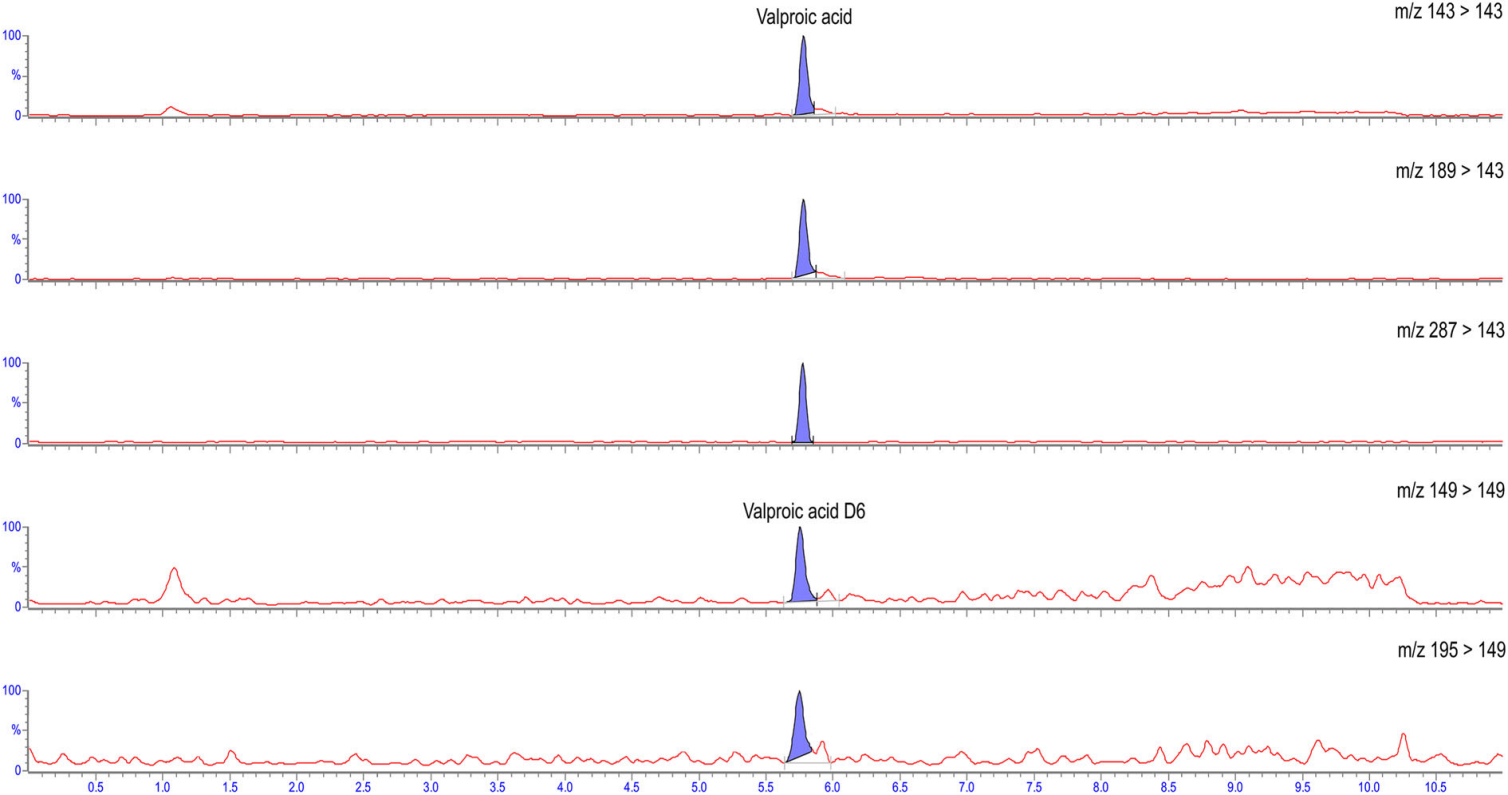
Extracted ion chromatogram (EIC) of parent ion (*m*/*z* 143/143), valproic acid adduct with formate (*m*/*z* 189/143), and valproic acid dimer (*m*/*z* 287/143) in sample (case 3): The calculated VPA concentration was 16.2 μg/ml. Internal standard (IS) was valproic acid‐D6 [Colour figure can be viewed at wileyonlinelibrary.com]

The selection of mobile phases composition allowed to work in an excess of formate ion without important variation during the gradient run. The MS source parameters were optimized in SCAN and product ion mode by infusing VPA at the concentration of 500 μg/ml. Source voltages and temperatures were intentionally selected to increase the signal of the mass‐to‐charge (*m*/*z*) ratio of the ions 287 and 189, respectively, corresponding to dimer VPA–VPA and formate–VPA adducts. Cone and capillary voltages were the parameters most affecting dimer and adduct intensity. The deuterated IS showed the same pattern of in source adducts formation (Figure 1). No other adducts were observed. Collision energies (CEs) were selected to increase intensity for transitions 287/143 and 189/143. No fragment was observed for the parent ion 143 for any of the tested CE, as already verified by other authors.^6^, ^9^, ^10^ Because the transition corresponding to mass‐to‐charge ratio 143/143 demonstrated to be the highest among the studied transitions, it was also added to the method and forwarded to validation. The ion signals used for quantification and qualification were selected according to ion intensities under the optimized MS conditions in standard solution: *m*/*z* 143/143 was considered the quantifier MRM transition, whereas *m*/*z* 189/143 and *m*/*z* 287/143 were selected as qualifiers. All ion signals were verified on whole blood for presence and abundance before method validation. The calculated surviving ion/secondary transitions ratios in matrix‐matched calibrators and quality controls were 1.5 and 9.3 for the adduct and the dimer, respectively. Reproducibility of the ratios was calculated over four non‐consecutive days during precision experiments and was considered acceptable (RSD 3% and 7%). Ratio tolerances for relative ion intensities followed the European Directive 96/23/EC concerning the performance of analytical methods,^18^ and thus, both transitions *m*/*z* 189/143 and *m*/*z* 287/143 were submitted to further method validation. Under the described conditions, VPA and IS were eluted in 5.78 and 5.75 min, respectively. Blank samples with and without IS showed no interferents. The LOD/LOQ values were 2 and 5 μg/ml, well below the therapeutic range for VPA. Calibration was set from 5 to 800 μg/ml obtaining good linearity (mean *R* ^2^ = 0.99) with a weighing factor 1/*x* ^2^. Precision was good with values ≤ 6% both intraday and interday. Accuracy, expressed as bias, was below 4%. The calculated matrix effect was considered negligible, with an average value of 108% over the entire range. Stability of the processed sample at 24 h was acceptable, with values comprised between 4 and 13%. (Table 2) The validation experiments demonstrated that analyte formate and dimer adduct possessed the necessary stability to be used as qualifiers and showed an interesting option to increase selectivity for VPA detection. A similar application was demonstrated valid for VPA‐acetate adducts in serum, but not yet for formate adducts nor dimer adducts in blood. As a final step, the method was successfully applied to 10 real forensic blood samples with results detailed in Table 3. Five subjects with no history of VPA consumption, tested as controls, resulted negative. VPA was detected in all cases, in which the clinical history reported a treatment with the drug, at concentrations ranged from < LOQ to 16.2 μg/ml (Figure 2). As can be noted, a discrepancy from therapeutic plasma/serum concentrations was observed, even in consideration of the plasma/blood ratio. These data were already observed in VPA analysis on post‐mortem blood samples, and stability of VPA in stored blood was considered the major issue.^19^ To overcome VPA stability issue, the alternative way of collection by means of dried blood spots had been investigated by some authors with promising results.^9^, ^20^, ^21^ Unfortunately, this modality of collection could not be exploited in our cases, but it represents an interesting further application of the present method. Moreover, in the future, the advantage of working with mobile phases commonly used for drug analysis in positive ion mode could productively be exploited by integrating the analysis by adding more anti‐epileptic drugs to the same run.

**TABLE 2:** Accuracy and precision of the method

QC level	Concentration (μg/ml)	Precision		Accuracy	Processed sample stability
		RSD intraday, n. 5 (%)	RSD interday n. 20 (%)	Bias n. 20 (%)	Deviation n. 5 (%)
Low	25	5	6	0.2	4
Intermediate	75	3	4	3	13
High	500	2	3	4	8

*Notes*: RSD = relative standard deviation; QC, quality control.

**TABLE 3:** Summary of the 10 real forensic cases

Case number	Clinical history	Type of forensic evaluation	VPA concentration (μg/ml)
Case 1	Treated with VPA	Deceased – judicial autopsy	5.9
Case 2	Treated with VPA	Deceased – judicial autopsy	6.8
Case 3	Treated with VPA	Deceased – judicial autopsy	16.2
Case 4	Treated with VPA	DUID	< LOQ
Case 5–10	No treatment	Deceased – judicial autopsy	ND

*Notes*: DUID, driving under the influence of drugs; ND, not detected; LOQ, limit of quantification; VPA, valproic acid.

## 4 CONCLUSIONS

The present method allowed for the rapid LC–MS/MS detection of VPA in blood by exploiting the ion signals of VPA‐formate adduct and VPA dimer as qualifiers. The possibility to use mobile phase‐ion adducts and VPA dimer was demonstrated to increase selectivity in case of forensic applications on whole blood. However, VPA stability in post‐mortem cases remains one of the main issues.

## Data Availability

Data available on request due to privacy/ethical restrictions.
